# Supplementary figures and images for: The protein interactome of Escherichia coli carbohydrate metabolism
Source: PLoS One. 2025 Feb 4;20(2):e0315240. doi: 10.1371/journal.pone.0315240 (PMC11793828; doi:10.1371/journal.pone.0315240)

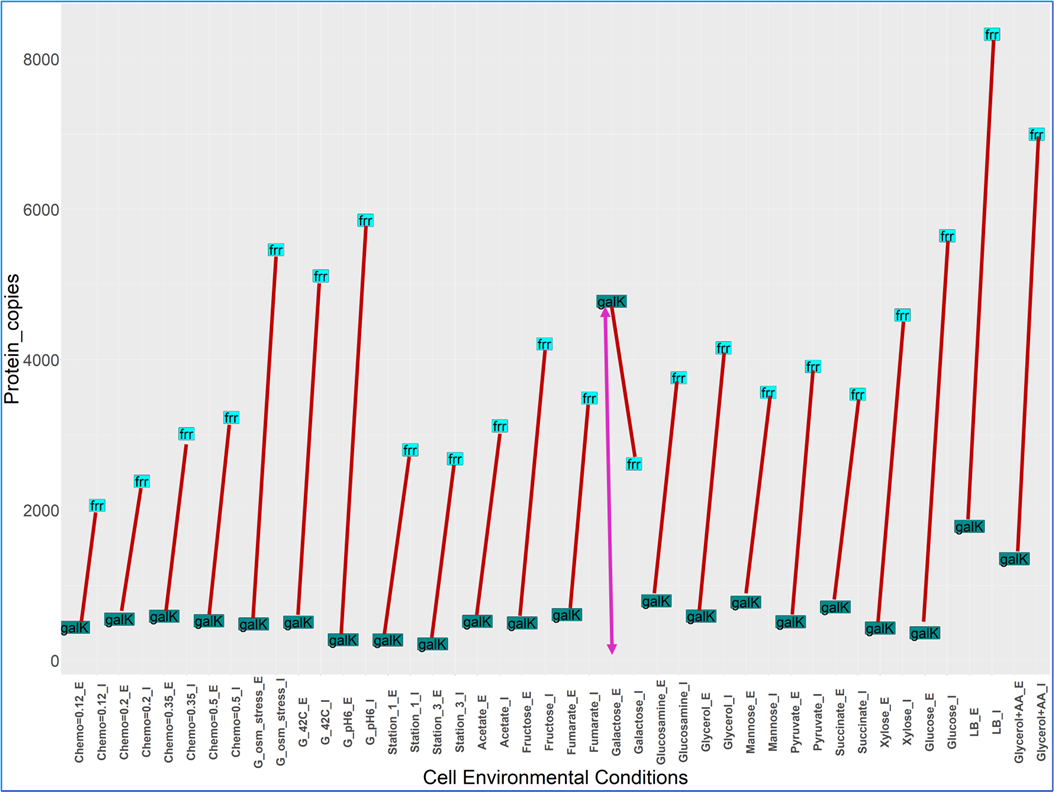

Supplement: S1 Fig — (TIF) [file pone.0315240.s001.tif]

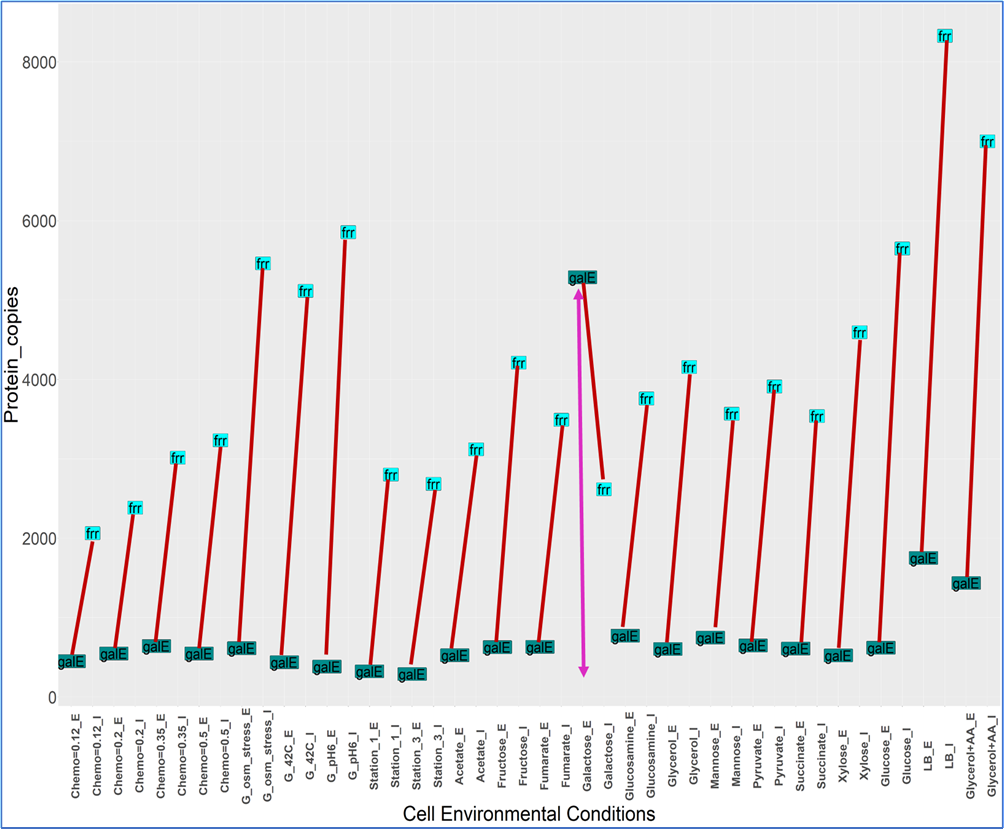

Supplement: S2 Fig — (TIF) [file pone.0315240.s002.tif]

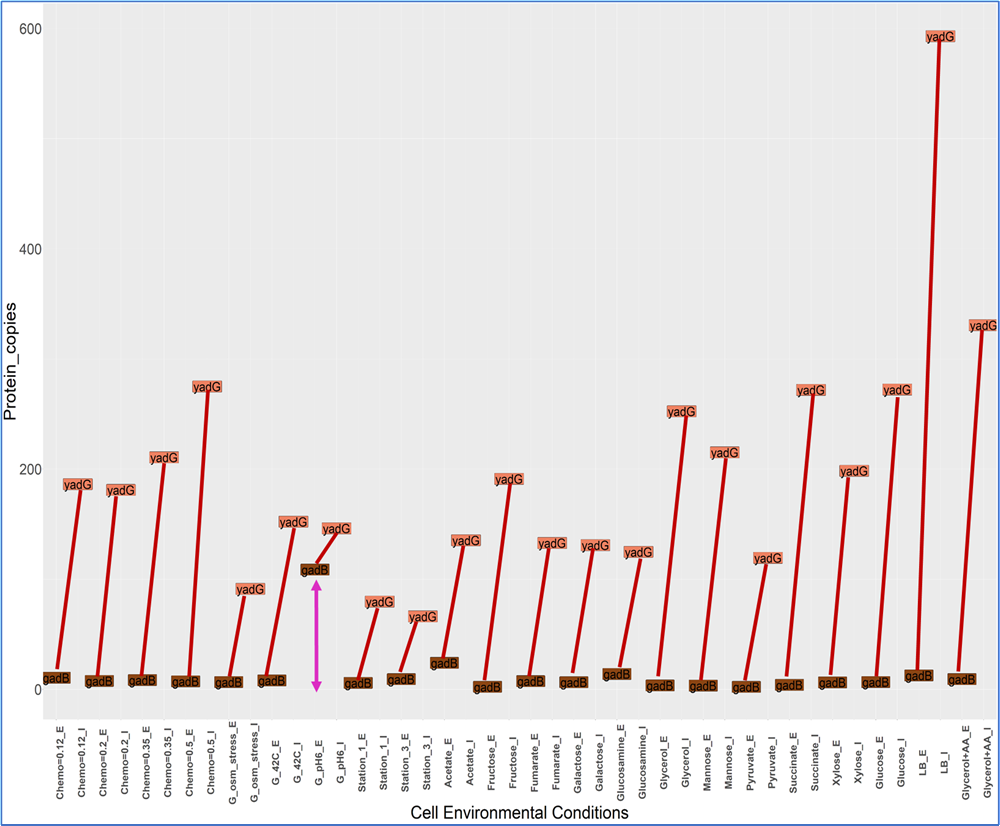

Supplement: S3 Fig — (TIF) [file pone.0315240.s003.tif]

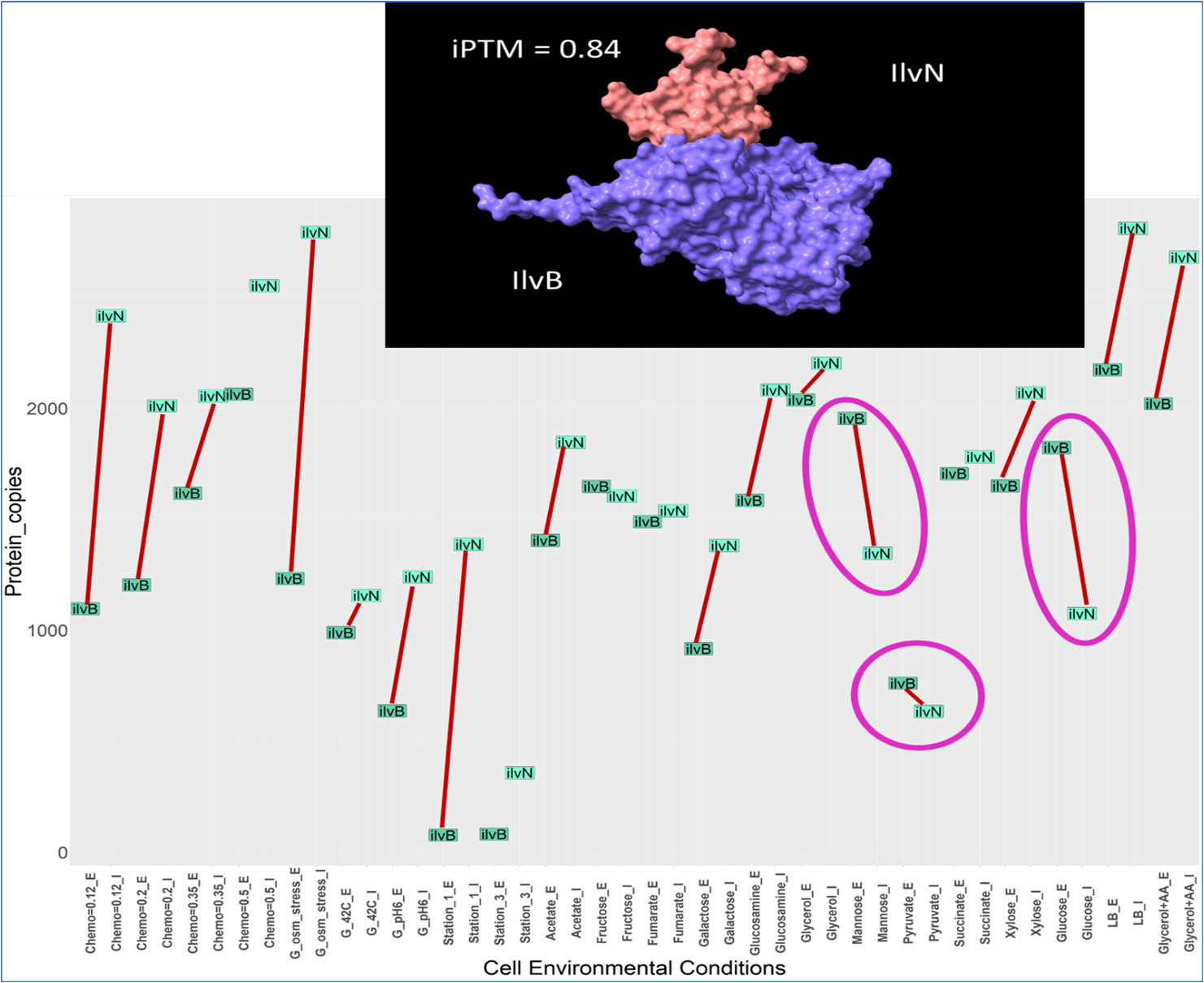

Supplement: S4 Fig — (TIF) [file pone.0315240.s004.tif]

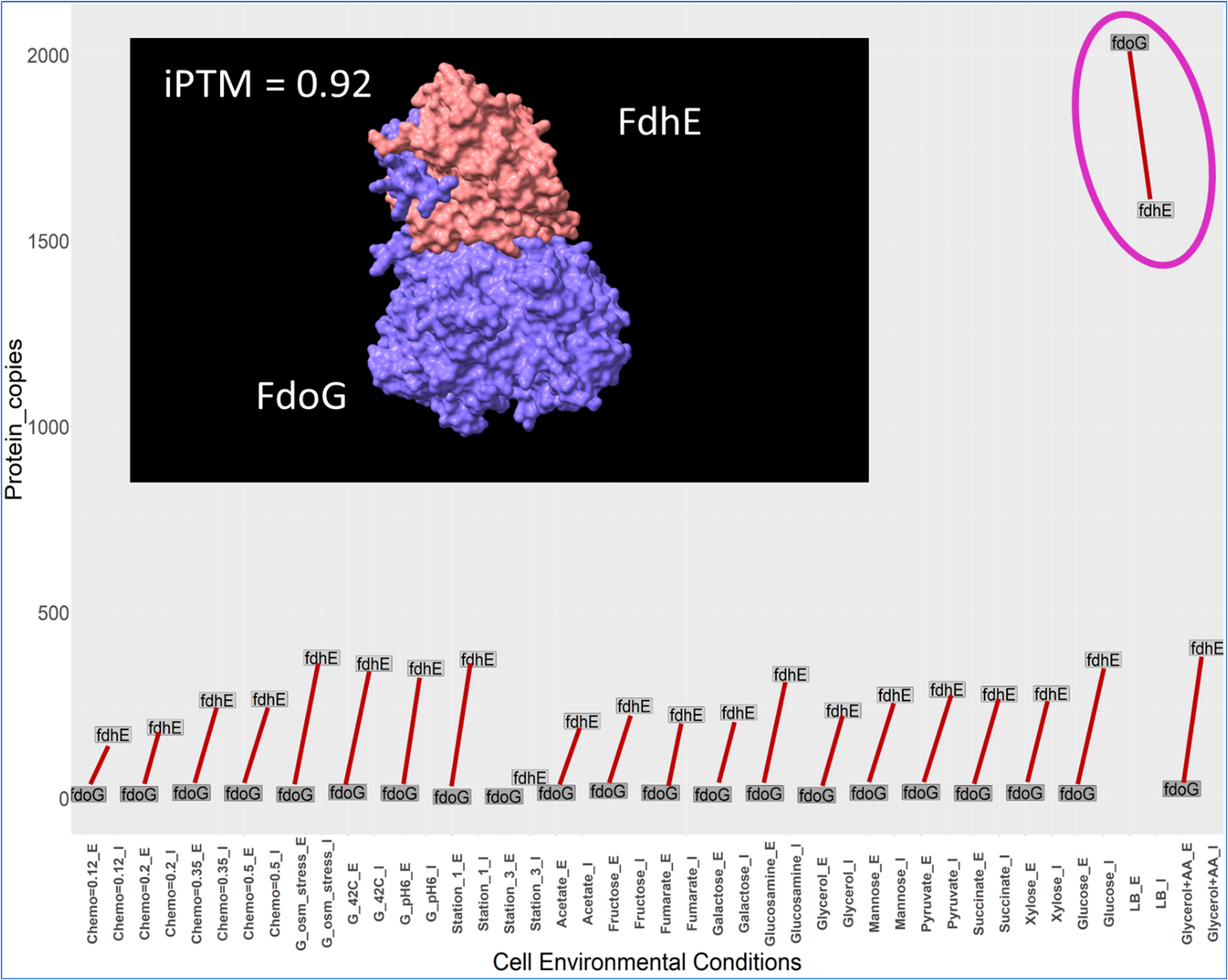

Supplement: S5 Fig — (TIF) [file pone.0315240.s005.tif]
