# Supplementary material for: The protein interactome of Escherichia coli carbohydrate metabolism
Source: PLoS One. 2025 Feb 4;20(2):e0315240. doi: 10.1371/journal.pone.0315240 (PMC11793828; doi:10.1371/journal.pone.0315240)
Supplement: Attachment — Submitted filename: Response to Reviewers.docx [file pone.0315240.s007.docx]

**Response to Reviewers**

We would like to thank the Reviewers for their careful reading and feedback on the manuscript. We appreciate the extensive suggestions on both the scientific findings and communication of results and have made extensive changes to the manuscript to incorporate the feedback from the Reviewers. Point-by-point responses to each Reviewer’s comments are included below.

**Reviewer 1:**

**Summary Comment:** However, I feel this is an incomplete and immature research and the manuscript is not well prepared.

**Response:** Based upon the comments of both Reviewers, extensive revisions have been made to the manuscript to improve both the language and organization of the manuscript and the scope of the research presented, including multiple new analyses.

We believe that the systematic analysis of protein-enzyme interactions for carbohydrate metabolism provides valuable insight into both the nature of these interactions and possible effects on metabolism. We agree that this area of study has not been explored much and there is much to be discovered, but given that we are among the few people who have investigated the problem discussed, it’s in the nature of the topic that it may appear immature.

**Summary Comment:** Moreover, I do not think this study provides sufficient new knowledge.

**Response:**

We respectfully disagree and believe that new knowledge has been found in several areas:

1) Interactor stoichiometry – By systematically studying protein-enzyme interactions across different conditions, interaction stoichiometries (with surprisingly large ranges) were characterized for the first time across a family of metabolic pathways.

2) Role of metabolic conditions – On top of the stoichiometric analysis, we show how different growth conditions affect interactions and thus regulation of metabolic pathways..

3) Conservation of interactions – We have shown that protein-enzyme interactions are conserved across thousands of bacterial genomes. While this is to be expected, we have produced a dataset for further analysis in other organisms.

4) Potential role of uncharacterized proteins – A number of metabolic enzymes were found to bind to uncharacterized proteins which should encourage the scientific community to take up more research in this direction of discovering novel metabolic as well as moonlighting functions of protein-enzyme interactions.

**Summary Comment:** Finally, I cannot agree that this study directly addresses the primary question of “how carbohydrate metabolism (CHM) in *E. coli* is regulated by their interaction properties with other proteins and their quantities” which they claimed in the Abstract and Conclusion.

**Response:** To better address this concern, we have revised both the Introduction as well as Results & Discussion. Analyses have been expanded to study the role of secondary interactions. We have decreased the threshold to identify more PEIs potentially impacting metabolism and thus, you will see 25 enzymes and 22 interactors potentially impacting metabolism (**Fig 9**) which was 20 and 19 in the previous version.

**The major comments of Reviewer 1 are highlighted in yellow in the manuscript with line numbers mentioned here.**

**Major Comment 1:**

**1.** However, I feel disappointed to find out that this study only generates a list of PEIs, which makes no clear difference from its sources IntAct database.

**Response:** While the IntAct database provided an initial list of interactions, these protein-enzyme interactions were reconciled with experimental data in different growth conditions to identify PEIs that are affected by different environments. Our results go way beyond the available data in IntAct (or Uniprot) and cannot be easily derived from any of these two databases.

**2.** Moreover, in the whole manuscript, I could feel the authors tried very hard to search for many works of literature to support or imply the potential of their findings related to PEIs.

**Response:** Yes, for an extensively studied organism like *E. coli* we believe that reconciling results with the existing body of research and knowledge is important even though it may seem redundant.

**3.** However, there is still nothing new from the PEIs in this study even if some literature had previously identified the same enzyme-protein interaction as their PEIs list.

**Response:** Although many PEIs found in our study had been known before from many small-scale studies, we demonstrated that such (potentially) regulatory interactions can be discovered on a large scale and thus much more efficiently, even though we concede that further studies are usually needed.

**4.** In the end, it turned out that the interpretation of the results/discussion looked like just a literature review.

**Response:** We thank the Reviewer for noticing the extensive inclusion of literature in the results and discussion as we believe that reconciling this new systematic analysis with the existing body of work on *E. coli* is critically important. The fact that the literature supports our results shows the significance and relevance of our results. However, in order to de-emphasize the literature review we have shortened the manuscript significantly and replaced some of that material by citations.

**Major Comment 2:** From the M/M to Results section, it looks like a direct copy and paste from a student thesis. The Results are fragmented and lacking clear logical connections. At least, each experiment or analysis should be composed of (1) scientific motivations/questions, (2) methodologies to investigate the issues (3) results, and (4) interpretations. Moreover, the figure legends are dispersed throughout the manuscript, and it is difficult to find them. It is disrespectful to reviewers who have to wade through it.

To me, the Discussion is relatively better in the scientific context. I would suggest rewriting a mixed Results/Discussion section.

**Response:** The original manuscript was submitted in conformity with the style and organization of the journal. Given the Reviewer’s comments, we have revised the manuscript to improve its readability and included the Figures in the manuscript and combined the Results & Discussion sections (**lines 175**)

**Major Comment 3:** It is confusing how to define and classify the “primary” and “secondary” interactors of the same enzyme/protein. Is it based on ratio or absolute abundance?

**Response:** The designation of primary or secondary interactor is not based on ratio or absolute abundance. We have explained what secondary interactors are and how they are different from primary interactors in **lines 358** to **373** and **Fig 11**.

**Major Comment 4:** Enzymes and interactors compete with other proteins to bind to each other”. Honestly, I am not convinced by simple discussions to claim that interactors compete with each other when only “predicted” overlapped binding sites are shown.

**Response:** In the revised paper, we have toned down the role of overlapping binding sites somewhat but we still believe that these predictions are valuable for further experimental testing (**lines 399-403 & Fig 11**). You will see some parts highlighted green as Reviewer 2 also requested some further explanation on this section.

**Major Comment 5:** *E. coli* protein-enzyme interactions may regulate carbohydrate metabolism”, I do not see any solid and direct evidence to support this conclusion solely according to the data generated in this study. The only way I can think of to improve this study is to perform some wet experiments to verify some previously unpublished and uninvestigated PEIs and then to prove that the interactor plays roles in the regulation of the paired enzyme.

**Response:** We fully agree that direct experimental validation is the gold standard and would allow for more detailed understanding of regulatory mechanisms. In lieu of new experimental data, our goal was to estimate the scale at which interactions are likely to have a physiological effect (and to use the existing literature to validate these estimates). As mentioned, the limitation of this study is its lack of experimental validations (see **lines 483-486).**

**The minor comments of Reviewer 1 are highlighted in blue in the manuscript with line numbers mentioned here.**

**Minor Comment 1:** Table 1: change “KEGG_Num” to “KEGG Identifier

**Response:** Done, **line 34** (**Table 1**)

**Minor Comment 2:** Intuitively, “non-metabolic enzyme” sounds make no sense to me. I assume all enzymes work for metabolisms of the organism. Please clarify this definition, and provide some examples showing an enzyme with non-metabolic functions.

Response: We have defined what non-metabolic enzymes are and gave examples of the same in **lines 196-199.**

**Minor Comment 3:** Please provide more detailed information in M/M for the “Conservation of PEIs” analysis. It is also good to know the classification of bacterial OG species used.

Response: We have provided more elaborate information in M/M for the Conservation of PEIs (**lines 117-123**).

**Minor Comment 4:** It would be also good to add one more column in Table S1 after “Enzyme” and “Interactor” to specify their functions, e.g., dehydrogenase, converting A from B, a regulator of enzyme X, etc.

**Response:** We have added columns of Protein Names, GO Molecular Function and GO Biological Process in **Table S1** for all the proteins and enzymes involved in PEIs. These three sets of information should describe what are their functions are, such as dehydrogenase, converting A from B, a regulator of enzyme X, etc.

**Minor Comment 5:** Number of PEIs scale with pathway size.” It is not surprising at all. PPI is universal. A bigger pathway composed of more enzymes of course gets more interactors. I will not feel this is an informative analysis unless “PEI density (numbers of interactors per enzyme)” is used for comparison and then some specific pathways are found to have unexpected higher or lower PEI density.

**Response:** We agree that a bigger pathway with more enzymes will bind more proteins, and it may seem not surprising (but we only knew after this had been tested). Therefore, as reviewer suggested we have added PEI density values and yes, we have got some pathways like pentose interconversion and inositol phosphate metabolism pathways having high PEI density values which was not expected as they are not central carbohydrate pathways (**lines 227-235** & **245-246**).

**Minor Comment 6:** The M/M for using ChimeraX and comparing overlapped binding sites is missing.

Response: We have added this in **lines 143-148**. You will see some green highlighted parts here as Reviewer 2 also demanded some explanation along these lines.

**Minor Comment 7:** Are the two interacting proteins' protein structures based on AlphaFold completely? In other words, are their interaction based on a predicted model or real protein crystal structures?

**Response:** The interacting protein structures are based on AlphaFold based software: ColabFold. Yes, they are based on predicted models as PDB structures of protein-protein interactions is rarely available (**lines 138-142**). You will see some green highlighted parts here as Reviewer 2 also demanded some explanation in this direction.

**Minor Comment 8:** Except for the galactose examples, how do you explain the other “inverse stoichiometry” cases?

**Response:** We have added a separate section discussing the inverse stoichiometry cases where either enzyme or interactor change their abundance but not both the proteins: **Section 3.6 (line 327).** We have also added a new example to these cases: Eno-GlpK.

**Minor Comment 9:** Fig S1-S5, what do “_E” ,“_I”, and “G_” stand for in the growth conditions X-axis?

**Response:** We have explained this now in the captions of **Fig 9, Fig 10 and Fig 12** and of **Fig S1-S5**.

**Minor Comment 10:** Fig S1-S5 needs to be re-prepared. Some gene name tags close to the Y-axis and right side border are truncated.

**Response:** We have solved this problem in not only **Fig S1-S5** but also in **Fig 9**, **Fig 10** and **Fig 12**.

**Minor Comment 11:** Move the statement “Schmidt et al [15] PEIs count = 179 in the figure to the figure legend or direct remove it.

Response: Done. We removed it from the figure and explained it in Figure caption. See **Fig 8**.

**Reviewer 2:**

**The major comments of Reviewer 2 are highlighted in green in the manuscript with line numbers mentioned here.**

**Major Concern 1:** The authors should justify the use of the IntAct database to retrieve PEIs as there are many PPI databases.

**Response:** The revised manuscript includes expanded clarification and explanation on the use of IntAct to retrieve PEIs but not other PPI databases in **(lines 80 – 83).**

**Major Concern 2:** The authors studies possible effects of secondary interactors under the assumption of competing roles. Secondary interactors could also facilitate the interaction of a PEI if they form stable multi-subunit complexes. This possibility should be mentioned.

**Response:** We agree that secondary interactors may not always compete but form stable protein complexes with PEIs. We mention that possibility in **lines 411-413**.

**Major Concern 3:** Details of how the contacts are calculated in Table 4 and Figure 6 should be given in materials and methods. Are they based on experimental structures or compute-modeled structures? If experimental structures were used, the PDB codes of the structures shown in Figure 6 should be given.

**Response:** We have explained the details of how contacts were calculated in **Table 5** and **Fig 11** now. You can find them in **lines 143-154**. The PDB structures, especially of protein-protein interactions are rarely available. All the structures are modeled in AlphaFold-based software ColabFold. You will see some blue highlighted parts here as Reviewer 1 also demanded some explanation in this direction.

**Major Concern 4:** While the secondary interaction and a PEI may share a certain fraction of contacting residues, they may not interfere with each other. Superposition of secondary interaction complex and the PEI complex to detect spatial clashes would be more suitable to infer competing roles of a secondary interactor.

**Response:** We agree that small overlaps between contact residues may not interfere with concomitant interactions but given that we have used predicted structures, it will difficult if not impossible to predict such effects, hence we consider them beyond the scope of this manuscript. However, we agree that only using the criteria of fraction of contact residues is not sufficient to explain the secondary interaction effects. Similarly, we have considered additional criteria such as Binding Energy but decided to use only Buried Surface Area in secondary interaction analysis in **Section 3.8 (lines 399-403 & Fig 11).** You will see some parts highlighted in yellow as Reviewer 1 also wanted some explanation along these lines.

**Major Concern 5:** The pLDDT score is residue based. Are the pLDDT scores in Figures 7 and 8 the average of all pLDDTs? pLDDT cannot be used to quantify the confidence of the complex. The authors can use AlphaFold3 to model the complex and use the PTM and iPTM scores as confidence measures of the overall complex and the interface respectively.

**Response:** We have replaced pLDDT scores with PTM and iPTM scores. We explain these in **lines 138-142.**

**The minor comments of Reviewer 2 are highlighted in pink in the manuscript with line numbers mentioned here.**

**Minor point 1:** *Escherichia coli*: change to italic. Line 22, Hpr (italic): change to regular font HPr.

**Response:** Changed now (**line 47**).

**Minor Point 2:** It is not clear what “non-spoke expanded interactions” means.

**Response:** We explain what it means in **lines 84 to L88**.

**Minor Point 3:** “PEIs in red in Table 5”. However, there is no red marking in Table 5.

**Response:** This mistake in the previous version has been corrected in the revised manuscript.

**Minor point 4:** To make the supplementary tables more informative, add gene names in Tables S1, S2 and S3 and add UniProt accessions in Table S4.

**Response:** We have combined all information in one **Table S1** now. We have even added some more information such as biological process, function etc. apart from gene and protein names.
